# The effect of donor–recipient sex matches on lung transplant survival: An analysis of the United Network for Organ Sharing database

**DOI:** 10.1016/j.xjon.2024.01.018

**Published:** 2024-02-07

**Authors:** Elizabeth Profozich, Abul Kashem, Roh Yanagida, Manish Suryapalam, Ke Cheng, Hiromu Kehara, Norihisa Shigemura, Yoshiya Toyoda

**Affiliations:** Division of Cardiovascular Surgery, Lewis Katz School of Medicine at Temple University, Philadelphia, Pa

**Keywords:** original manuscript, lung transplantation, donor–recipient matches, sex matching

## Abstract

**Objective:**

To investigate the impact of donor–recipient (DR) sex matches on survival after lung transplantation while controlling for size difference in the United Network of Organ Sharing (UNOS) database.

**Methods:**

We performed a retrospective study of 27,423 lung transplant recipients who were reported in the UNOS database (January 2005-March 2020). Patients were divided into groups based on their respective DR sex match: male to male (MM), male to female (MF), female to female, (FF), and female to male (FM). Kaplan–Meier curve and Cox regression with log-rank tests were used to assess 1-, 3-, 5-, and 10-year survival. We also modeled survival for each group after controlling for size-related variables via the Cox regression.

**Results:**

Kaplan–Meier curves showed overall significance at 1-, 3-, 5-, and 10-year end points (*P* < .0001). Estimated median survival time based on Kaplan–Meier analysis were 6.41 ± 0.15, 6.13 ± 0.18, 5.86 ± 0.10, and 5.37 ± 0.17 years for FF, MF, MM, and FM, respectively (*P* < .0001). After we controlled for size differences, FF had statistically significantly longer 5- and 10-year survival than all other cohorts. MF also had statistically significantly longer 5- and 10-year survival than FM.

**Conclusions:**

When variables associated with size were controlled for, FF had improved survival than other DR groups. A female recipient may experience longer survival with a female donor’s lungs versus a male donor’s lungs of similar size.

## Graphical abstract

**Figure:**
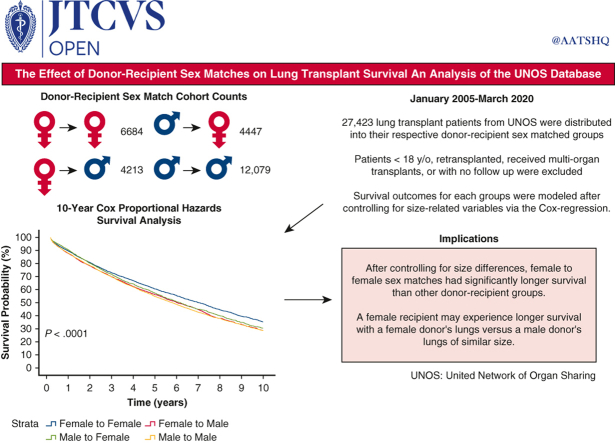

**Figure:**
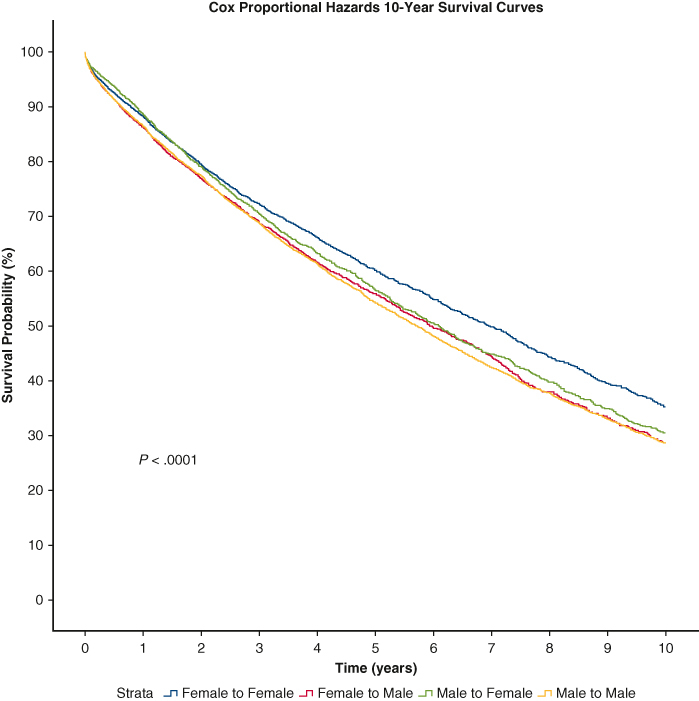
Predicted survival after Cox regression analysis between DR sex-matched groups.

**Central Message**

Donor–recipient (DR) sex matching significantly impacts survival post-lung transplantation. After controlling for DR size, female to female matches have increased survival.

**Perspective**

The impact of donor–recipient (DR) matching on survival in lung transplantation is of epidemiological interest. The impact of DR sex matching is understudied, and results are conflicting. When there is a significant finding, it is often attributed to DR size difference. In this study, we assessed survival differences between DR sex-matched groups while controlling for DR size.

Donor–recipient (DR) matching in lung transplantation is a complicated process that is based on factors including blood group compatibility, immune system matching, and size.^1^ Certain factors, such as biological sex, may have an effect on survival but are not included in this algorithm. Although adding more qualifiers to match donors to recipients has the potential to decrease the number of suitable matches for a life-saving procedure, it is of epidemiologic interest to explore how factors excluded from the matching process affect survival.

There is currently limited research on the survival of DR biological sex-matched pairs after lung transplantation, and the available research is predominantly conflicting. Previous studies have shown improved survival associated with male donors and female recipients.^2^, ^3^, ^4^, ^5^ In contrast, studies have also found no correlation between donor or recipient sex or DR sex combination on survival.^6^, ^7^, ^8^ When a correlation is found, researchers frequently attribute difference in survival to sex-associated size differences.

It remains unclear whether DR sex matching truly has an effect on recipient survival. In addition, if there is a survival difference between DR sex-matched groups, it is unknown whether there would still be a difference after controlling for donor and recipient size differences.

The aim of this study was to analyze the effect of DR sex matching on long-term survival post-lung transplantation using the United Network for Organ Sharing (UNOS) database, as well as model survival in these groups after controlling for DR size differences.

## Methods

### Data Collection and Study Population

This study was a retrospective analysis performed using transplant data from the UNOS database, a comprehensive database containing information on all patients who undergo lung transplantation in the United States. The study population was DR matches between January 2005 and March 2020. Patients were separated into 4 cohorts by sex of donor and recipient: male to male (MM), male to female (MF), female to female (FF), and female to male (FM). All patients younger than 18 years of age, patients with no follow-up, patients receiving multiorgan transplants, and patients who were retransplanted were excluded from this analysis.

### Statistical Analysis

All the data were analyzed using SPSS 26.0 (IBM Corp). The Kaplan–Meier curve was drawn in R (version, 4.3.0; R Foundation for Statistical Computing). Many demographic variables and baseline characteristics of lung transplant donors and recipients were noted and expressed as mean ± standard deviation. Factors assessed included age, height, weight, body mass index (BMI), and race, as these demographic variables have association with donor and recipient size. The nominal variables were evaluated for significance using Pearson’s χ^2^ test. For continuous variables, a Shapiro–Wilk test was used to assess normality, and an *F* test was used to assess homogeneity of variance. In cases of non-normality, a Kruskal–Wallis H-test was performed to evaluate significance in distribution. Otherwise, an analysis of variance test was performed. For further pairwise comparisons, a Mann–Whitney *U* test was performed in cases of non-normality, and a 2-sample *t*-test was used in cases of normality. If the *F*-test showed nonsignificance, then the *t*-test was performed with an equal variance assumption. If the *F*-test showed significance, a 2-sample *t*-test with unequal variances was used.

Survival was assessed using Kaplan–Meier survival curves with log-rank test to assess significance at 1-, 3-, 5-, and 10-years posttransplant by sex-matched cohort. Both overall significance and pairwise significances were assessed.

We also chose to perform regression modeling using stratified Cox proportional-hazards to evaluate the impact covariates on survival by age cohort. Survival at 5- and 10-years’ posttransplantation were evaluated.

The Cox regression model assumes that the effects of covariates on survival are not time dependent. To test whether this assumption was met, a hierarchical regression strategy was used. First, a Cox regression was individually performed with the original set of covariates. Then, a potential time-dependent interaction with each covariate was modeled, and the change in fit between the original model and interaction model was evaluated using a likelihood ratio test for statistical significance. Verifying that there is no time-dependent interaction provides especially strong statistical evidence for derived conclusions on survival.

## Results

### Demographics

Study demographics are displayed in Table 1. Of the 27,423 patients who received lung transplants from January 2005 to March 2020, the largest demographic represented in the sample were White biological male patients (50.0%). All perioperative factors showed overall significance between cohorts as well as pairwise significance between each cohort.

**Table 1:** Donor–recipient sex match group demographics

Demographic variables	Total	MM	MF	FF	FM	*P* value
Count	27,423	12,079	4447	6684	4213	
Recipient age, y	56.0 ± 13.0	57.4 ± 12.3	53.9 ± 13.5	53.5 ± 13.7	57.9 ± 12.4	**<.001**
Recipient height, cm	170.0 ± 9.9	176.7 ± 7.1	163.5 ± 6.8	160.4 ± 6.3	172.5 ± 7.1	**<.001**
Recipient weight, kg	73.4 ± 16.7	80.7 ± 15.6	64.4 ± 13.7	63.6 ± 13.7	77.9 ± 14.8	**<.001**
Recipient BMI, kg/m^2^	25.3 ± 4.6	25.8 ± 4.4	24.1 ± 4.7	24.6 ± 4.9	26.1 ± 4.3	**<.001**
Donor age, y	34.7 ± 14.1	32.5 ± 13.4	31.6 ± 13.9	38.5 ± 14.3	38.4 ± 13.8	**<.001**
Donor height, cm	171.7 ± 10.2	178.4 ± 7.4	172.8 ± 9.0	162.2 ± 7.2	166.2 ± 6.6	**<.001**
Donor weight, kg	77.1 ± 17.7	82.1 ± 16.7	76.1 ± 16.8	70.5 ± 17.3	74.1 ± 17.8	**<.001**
Donor BMI, kg/m^2^	26.1 ± 5.4	25.8 ± 4.8	25.4 ± 4.9	26.8 ± 6.2	26.8 ± 6.1	**<.001**
Transplant type						**<.001**
SLT	8448 (30.8%)	4024 (33.3%)	1306 (29.4%)	1771 (26.5%)	1347 (32.0%)	
DLT	18,975 (69.2%)	8055 (66.7%)	3141 (70.6%)	4913 (73.5%)	2866 (68.0%)	
Length of stay, d	25.8 ± 30.7	24.3 ± 28.5	25.6 ± 30.9	27.1 ± 31.5	28.2 ± 34.9	**<.001**
Recipient race						**<.001**
White	22,501 (82.1%)	10,383 (86.0%)	3662 (82.3%)	5138 (76.9%)	3318 (78.8%)	
Black	2428 (8.9%)	843 (7.0%)	465 (10.5%)	839 (12.6%)	281 (6.7%)	
Hispanic	1828 (6.7%)	628 (5.2%)	239 (5.4%)	519 (7.8%)	442 (10.5%)	
Other	666 (2.4%)	225 (1.9%)	81 (1.8%)	188 (2.8%)	172 (4.1%)	

Each variable is displayed as mean ± standard deviation. Pairwise significance was calculated overall between donor–recipient groups for each variable. *P* values in bold are statistically significant. *MM*, Male to male; *MF*, male to female; *FF*, female to female; *FM*, female to male; *BMI*, body mass index; *SLT*, single-lung transplant; *DLT*, double-lung transplant.

### Survival Analysis via Kaplan–Meier Curve and Cox Proportional-Hazards Regression

**Figure 1:**
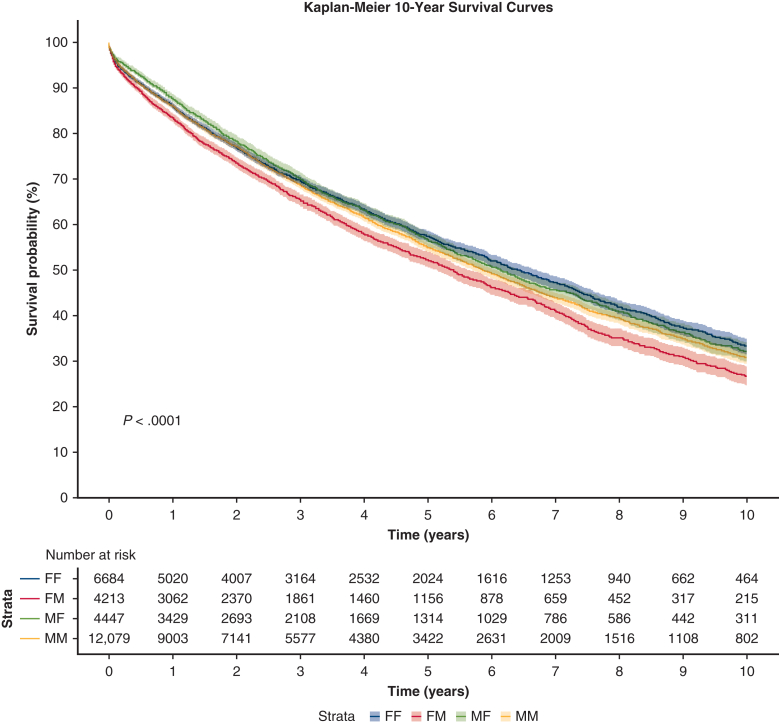
Donor–recipient sex match effect on survival. Kaplan–Meier survival curves show a statistically significant difference in 10-year survival based on donor–recipient sex-matched groups (*P* < .0001). 95.0% confidence limits are displayed as *shading*. *FF*, Female to female; *FM*, female to male; *MF*, male to female; *MM*, male to male.

Kaplan–Meier curves showed overall significant difference in survival at 1-, 3-, 5-, and 10-year end points (*P* < .0001). Figure 1 shows a statistically significant difference in 10-year survival based on DR sex-matched groups (*P* < .0001). FM had significantly lower survival compared with all other DR sex-matched groups at 1-, 3-, 5-, and 10-year time points (*P* < .001 for all). Pairwise assessment showed MF survived longer than MM and FF at 1 year (*P* = .003, *P* = .02, respectively); however, this relationship was not present at subsequent time points. FF survived longer than MM only in the 10-year analysis (*P* = .01).

Estimated median survival time based on Kaplan–Meier analysis was 6.41 ± 0.15 years, 6.13 ± 0.18 years, 5.86 ± 0.10 years, and 5.37 ± 0.17 years for FF, MF, MM, and FM, respectively (*P* < .0001). In clinical terms, estimated median survival time was about 6 years and 5 months for FF, 6 years and 1.5 months for MF, 5 years and 10 months for MM, and 5 years and 4.5 months for FM.

Cox proportional-hazards regression was performed for 5- and 10-year survival for each variable, and results are shown in Tables 2 and 3, respectively. For 5-year survival regression, recipient weight (hazard ratio [HR], 1.013; 95% confidence interval [CI], 1.000-1.027; *P* = .05), recipient BMI (HR, 0.960; 95% CI, 0.925-0.997; *P* = .04), donor age (HR, 1.003; 95% CI, 1.001-1.004; *P* < .0001), donor height (HR, 0.990; 95% CI, 0.980-0.999; *P* = .04), double-lung transplant (HR, 0.725; 95% CI, 0.692-0.759; *P* < .0001), and length of stay (HR, 1.007; 95% CI, 1.007-1.008; *P* < .0001) were significant. Specifically, lower recipient weight, greater recipient BMI, younger donor age, taller donor height, double-lung transplant, and shorter length of stay were associated with longer survival.

**Table 2:** Cox proportional hazards 5-year survival regression

Demographic variables	Coefficient	95.0% CI	*P* value
Recipient age	1.002	1.000-1.003	.11
Recipient height	0.989	0.977-1.001	**.04**
Recipient weight	1.013	1.000-1.027	.06
Recipient BMI	0.960	0.925-0.997	**.045**
Donor age	1.003	1.001-1.004	**<.001**
Donor height	0.990	0.980-0.999	.60
Donor weight	1.002	0.992-1.013	.04
Donor BMI	0.992	0.962-1.023	.66
Single vs double	0.725	0.692-0.759	**<.001**
Length of stay	1.007	1.007-1.008	**<.001**
Recipient race: White			.15
vs Black	1.009	0.937-1.086	.82
vs Hispanic	0.929	0.849-1.017	.11
vs Other	0.884	0.767-1.018	.09

Cox proportional-hazards 5-year survival regression was calculated for each variable. *P* values in bold are statistically significant. *CI*, Confidence interval; *BMI*, body mass index.

**Table 3:** Cox proportional-hazards 10-year survival regression

Demographic variables	Coefficient	95.0% CI	*P* value
Recipient age	1.005	1.003-1.007	**<.001**
Recipient height	0.990	0.980-1.001	.07
Recipient weight	1.011	1.000-1.023	.06
Recipient BMI	0.967	0.935-1.000	.05
Donor age	1.003	1.002-1.005	**<.001**
Donor height	0.992	0.983-1.000	.06
Donor weight	1.003	0.993-1.012	.59
Donor BMI	0.993	0.966-1.020	.60
Single vs double	0.691	0.664-0.720	**<.001**
Length of stay	1.007	1.007-1.007	**<.001**
Recipient race: White			.13
vs Black	0.998	0.934-1.066	.96
vs Hispanic	0.921	0.848-0.999	**.048**
vs Other	0.913	0.805-1.036	.16

Cox proportional-hazards 10-year survival regression was calculated for each variable. *P* values in bold are statistically significant. *CI*, Confidence interval; *BMI*, body mass index.

For 10-year survival regression, recipient age (HR, 1.005; 95% CI, 1.003-1.007; *P* < .0001), donor age (HR, 1.003; 95% CI, 1.002-1.005; *P* < .0001), double-lung transplant (HR, 0.691; 95% CI, 0.664-0.720; *P* < .0001), length of stay (HR, 1.007; 95% CI, 1.007-1.007; *P* < .0001), and Hispanic race (HR, 0.921; 95% CI, 0.848-0.999; *P* < .05) were significant. Specifically, younger recipient age, younger donor age, double-lung transplant, shorter length of stay, and Hispanic race were associated with longer survival.

### Predicted Survival Outcomes via Cox Proportional-Hazards Model

**Figure 2:**
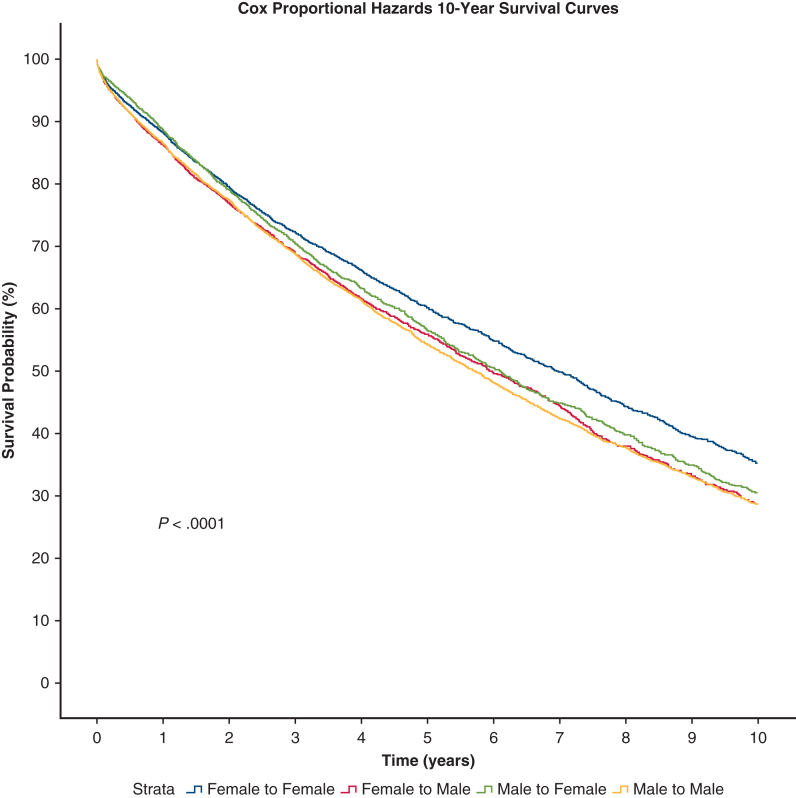
Donor–recipient sex match effect on survival after Cox regression analysis. Cox proportional-hazards survival analysis shows a statistically significant difference in 10-year survival based on donor–recipient sex-matched groups (*P* < .0001).

Ten-year predicted survival curves after controlling for variables correlated with size (recipient height, recipient weight, recipient BMI, donor height, donor weight, and donor BMI) via Cox regression are displayed in Figure 2. Cox proportional-hazards survival analysis showed a statistically significant difference in 10-year survival based on DR sex-matched groups (*P* < .0001). Pairwise testing between DR sex-matched groups at 5 and 10 years is displayed in Table 4. At 5 years, pairwise testing showed FF matches had longer predicted survival than MM, FM, and MF (*P* < .001, *P* < .001, and *P* = .01, respectively). MF had longer predicted 5-year survival than FM and MM (*P* = .002, *P* < .001, respectively). There was no significant difference in predicted 5-year survival between MM versus FM (*P* = .21). For 10-year survival, pairwise testing showed FF matches had longer predicted 10-year survival than MM, FM, and MF (*P* < .001 for all). MF had longer predicted 10-year survival than FM (*P* = .02). There was no significant difference between MM versus FM (*P* = .67) and MM versus MF (*P* = .07).

**Table 4:** Pairwise comparisons 5- and 10-year survival table

DR group	5-y			10-y		
	Coefficient	95.0% CI	*P* value	Coefficient	95.0% CI	*P* value
MM vs FF	0.804	0.742-0.871	**<.001**	0.841	0.784-0.903	**<.001**
MM vs FM	0.957	0.894-1.025	.21	0.987	0.928-1.049	.67
MM vs MF	0.889	0.826-0.956	**<.001**	0.927	0.868-0.989	.07
FF vs FM	1.191	1.103-1.285	**<.001**	1.173	1.096-1.255	**<.001**
FF vs MF	1.105	1.027-1.190	**.01**	1.101	1.032-1.175	**<.001**
FM vs MF	0.889	0.826-0.956	**<.001**	0.927	0.868-0.989	**.02**

Pairwise comparisons at 5- and 10-year survival were calculated between each cohort. *P* values in bold are statistically significant. *CI*, Confidence interval; *MM*, male to male; *FF*, female to female; *FM*, female to male; *MF*, male to female.

## Discussion

In this study, we reviewed the impact of DR sex matching on survival post-lung transplantation while controlling for demographic variables related to DR size differences in patients from the UNOS database. Our analysis revealed the following: (1) DR sex-matched groups had significantly different 1-, 3-, 5-, and 10-year survival, with FM having significantly lower survival than all other groups at each time point; and (2) after we controlled for size-related factors via Cox regression, the survival difference between MM and FM groups was no longer significant and the FF group had increased predicted survival compared with all other DR sex-matched groups.

Not only are previous studies investigating DR sex matches conflicting, but there are also few studies that analyzed survival between individual DR sex match groups. Most studies analyzed survival based on donor sex and recipient sex separately.^2^^,^^4^^,^^7^ Our study aimed to provide survival information for each DR sex-matched cohort and pairwise analysis to develop more detailed results. There has not been a study addressing this topic that takes an approach using as large of a sample size as the one used in our study with the UNOS database. We used Cox regression analysis to control for size differences and modeled new survival curves to determine whether sex matching affects survival.

Initial Kaplan–Meier analysis showed overall significance between DR sex-matched cohorts at each end point discussed. FF had the longest overall survival, followed by MF, MM, and FM had the worst outcomes. Multiple studies have found similar results, including Demir and colleagues,^9^ who demonstrated female recipients to have superior survival irrespective of donor sex and found FM to have the worst survival in their single-center retrospective study. A similar trend of superior survival in female recipients has been documented in another single-center analysis by Alvarez and colleagues.^2^ In a 2006 analysis of the International Society of Heart and Lung Transplantation Registry, Sato and colleagues^10^ also found FM to have greater 90-day mortality and FF to have lower 90-day mortality compared with the MM reference category.

After we controlled for variables correlated with size (recipient height, recipient weight, recipient BMI, donor height, donor weight, and donor BMI), via Cox regression, predicted 10-year survival curves still showed overall significance between DR sex-matched cohorts, which confirms that sex match affects survival independent of size difference. The markedly worse survival of the FM group compared with other DR sex-matched groups that is apparent in the Kaplan–Meier curve is not present in the Cox regression survival model. Thus, the shorter survival for FM observed in clinical could be explained by sex-associated size differences.

Female recipient advantage may be due to overall age differences in our female and male recipient cohorts. The mean age of male recipients was older than the mean age of female recipients, which may explain shorter survival in male recipients (58 vs 54 years). Interestingly, the mean age of male donors appears to be younger than the mean age of female donors (32 vs 38 years), so the 4-year difference between sexes in recipients could be offset by the 6-year age difference between sexes in donors. Another hypothesis could be that the female survival is due to lower incidence of chronic lung allograft dysfunction, which is a major source of death after lung transplantation. According to the International Society for Heart and Lung Transplant registry, 41.4% of recipients develop bronchiolitis obliterans syndrome (the predominant phenotype of chronic lung allograft dysfunction [CLAD]) by 5 years after lung transplant.^11^ A lower incidence of CLAD could explain the survival advantage in female patients that is more prominent earlier on.

Lung transplantation improves survival for people with end-stage lung disease regardless of DR sex matching, and optimal sex matching should not be a necessary variable to approve a match. However, these findings shed light on the survival differences that we observe between DR pairs and provide a basis to continue further research on survival prolongation post-lung transplantation.

Our study is limited by several factors that are unavoidable in a large, multicenter study. Primarily, we are limited by missing data in UNOS. Our study also forfeits the benefits that come with a single-center prospective study; however, by using the UNOS database, we were able to observe a larger pool of patients. Finally, there were also variables of interest that we were unable to use in our analysis (CLAD status and indication for transplant, for example) because they were either not present in the UNOS database, or there were too many different responses to meaningfully compare.

## Conclusions

In conclusion, our analysis of patients after lung transplant from the UNOS database demonstrated that DR sex matching has an impact on survival after size-related variables are controlled for. When variables associated with size were controlled for, survival of FF was greater than other DR groups. Thus, female recipients may experience longer survival with a female donor’s lungs versus a male donor’s lungs of similar size.

## Conflict of Interest Statement

Y.T. reported clinical research and grant support from Transmedics Incorporated, Cerus Corporation ReciPe Study, and EvaHeart Incorporated. This had no relationship with the current study and has not affected the integrity of our report. All other authors reported no conflicts of interest.

The *Journal* policy requires editors and reviewers to disclose conflicts of interest and to decline handling or reviewing manuscripts for which they may have a conflict of interest. The editors and reviewers of this article have no conflicts of interest.
